# Human milk extracellular vesicle miRNA expression and associations with maternal characteristics in a population-based cohort from the Faroe Islands

**DOI:** 10.1038/s41598-021-84809-2

**Published:** 2021-03-12

**Authors:** Allison Kupsco, Diddier Prada, Damaskini Valvi, Lisa Hu, Maria Skaalum Petersen, Brent Coull, Philippe Grandjean, Pal Weihe, Andrea A. Baccarelli

**Affiliations:** 1 Department of Environmental Health Sciences, Columbia University Mailman School of Public Health, New York, NY 10023 USA; 2 Unit for Biomedical Research in Cancer, Instituto Nacional de Cancerologia, Universidad Nacional Autonoma de Mexico, 14080 Mexico City, Mexico; 3 Department of Environmental Medicine and Public Health, Icahn School of Medicine at Mount Sinai, New York, NY 10029 USA; 4 Department of Occupational Medicine and Public Health, The Faroese Hospital System, Tórshavn, Faroe Islands; 5 Center of Health Science, University of the Faroe Islands, Tórshavn, Faroe Islands; 6 Department of Environmental Health, Harvard T.H. Chan School of Public Health, Boston, MA USA; 7 Department of Environmental Medicine, University of Southern Denmark, Odense C, Denmark

**Keywords:** miRNAs, Extracellular signalling molecules, Biomarkers

## Abstract

Human milk plays a critical role in infant development and health, particularly in cognitive, immune, and cardiometabolic functions. Milk contains extracellular vesicles (EVs) that can transport biologically relevant cargo from mother to infant, including microRNAs (miRNAs). We aimed to characterize milk EV-miRNA profiles in a human population cohort, assess potential pathways and ontology, and investigate associations with maternal characteristics. We conducted the first study to describe the EV miRNA profile of human milk in 364 mothers from a population-based mother-infant cohort in the Faroe Islands using small RNA sequencing. We detected 1523 miRNAs with ≥ one read in 70% of samples. Using hierarchical clustering, we determined five EV-miRNA clusters, the top three consisting of 15, 27 and 67 miRNAs. Correlation coefficients indicated that the expression of many miRNAs within the top three clusters was highly correlated. Top-cluster human milk EV-miRNAs were involved in pathways enriched for the endocrine system, cellular community, neurodevelopment, and cancers. miRNA expression was associated with time to milk collection post-delivery, maternal body mass index, and maternal smoking, but not maternal parity. Future studies investigating determinants of human EV-miRNAs and associated health outcomes are needed to elucidate the role of human milk EV-miRNAs in health and disease.

## Introduction

Human milk plays a critical role in infant development and health, as it provides crucial nutrients to the newborn including essential vitamins, minerals, proteins, lipids, and carbohydrates^1^. Therefore, human milk can influence development of the child brain, gut, metabolism, and immune system in the long-term programming of child physiology and health^2^. Within the last 10 years, research has suggested that other bioactive compounds in milk, such as nucleic acids, can be transferred from mother to child, with the potential to influence child development^3^. Many of these compounds may be contained within extracellular vesicles (EVs).

EVs are small, secreted, membrane-enclosed vesicles that can originate from a variety of tissues and are found in most human biofluids^4,5^. Generally thought to be composed of exosomes, microvesicles, and apoptotic bodies, EVs are highly heterogenous and vary greatly in origin, size, and content. Microvesicles range in size between 50 and 1000 nm and are released via blebbing of the plasma membrane, whereas exosomes are 30 to 150 nm in size and produced from the endosomal network to be released into biofluids from multivesicular bodies. Apoptotic bodies are large vesicles typically greater than 1000 nm that are created by blebbing of the plasma membranes from apoptotic cells. In the current analysis, we consider microvesicles and exosomes as EVs, as they are actively released from live cells. Milk EVs are released by the luminal epithelium and have been demonstrated to survive digestion in vitro and may be taken up by recipient cells in the infant^6–10^. These milk EVs can then induce changes in gene expression in vitro^6^, induce systemic metabolic alterations in vivo^9^, and may play a role in newborn immunity^11–13^.

One of the primary biological components of EVs are microRNAs (miRNAs)^14^. miRNAs are small, 22 nucleotide (nt) long, non-coding RNA molecules that have emerged as key regulators of gene expression via the repression or degradation of mRNA transcripts^15^. MiRNAs precisely regulate gene expression^16,17^ to control the cellular fate, differentiation, and stress response^18^. Through this mechanism, they play a role in numerous biological processes, from cell development to death and disease^19^. MiRNAs are differentially sorted into EVs by the cell^20^, suggesting that they are intentional messages sent from one cell to another. This indicates that the miRNA cargo of human milk EVs have the potential to serve as a mechanistic biological marker of maternal-child communication, potentially associated with external (e.g. environmental exposures^21^) or internal (e.g. maternal stress or maternal diseases) factors.

To date a few studies have examined human milk miRNA expression, reporting a large miRNA number and diversity. These studies have laid the foundation for the current work, demonstrating that miRNA profiles differ across lactation^22–25^, from other biofluids^26^, and by milk fraction (e.g. fat vs skim)^22,25,27^. For example a study comparing miRNAs isolated from human milk lipids and cells isolated at 2 months, 4 months, and 6 months post-delivery identified a large number of miRNAs in common between stages, but found that expression levels of approximately 200 (25–30%) miRNAs differed between stages with greatest upregulation at month 4^22^. Another study comparing cell-free miRNAs in plasma and human colostrum found 308 miRNAs differentially expressed between them^26^.

However, few of these studies have investigated human milk miRNAs within EVs, which may be the only biological cargo to survive digestion in the infant^7,13,24^. EV-miRNAs analysis has been shown to provide a more sensitive and reliable signal than miRNA extraction from crude biofluids^28^. Nonetheless, studies that have investigated human milk EV-bound miRNAs have been limited in population size (from N = 4 to 54 participants), with most only examining a few milk samples and even fewer have formally examined the associations of these miRNAs with maternal characteristics^7,13,24,29^. Therefore, we conducted the first study to characterize human milk EV-miRNAs from a large human population study (N = 364). The objectives of this analysis were to: (1) determine expression of human milk EV-miRNAs in a larger population (N = 364) compared to previous studies; (2) characterize clusters of miRNAs by expression levels; (3) characterize the potential biological functions of these miRNAs using gene ontology; and (4) examine associations of miRNAs with maternal body mass index (BMI), smoking, parity, and collection date.

## Results

### Study population characteristics

Participating mothers had a mean age of 29.8 ± 5.0 years at delivery, a mean pre-pregnancy BMI of 23.9 ± 3.85 kg/m^2^, and 26.6% reported to have smoked during pregnancy (Table 1). The majority were multiparous (76.1%) with 9.1% were diagnosed with gestational diabetes and 1.6% with preeclampsia. The majority delivered with a vaginal birth, while 10.2% delivered via C-section and 3% via vacuum extraction. Approximately half of children born were male.

**Table 1:** Characteristics of study population (n = 364).

Maternal characteristics	Mean ± SD
Maternal age (years)	29.3 ± 4.95
Maternal pre-pregancy BMI (kg/m^2^)	23.9 ± 3.85
Weight gain during pregnancy (kg)	15 ± 5.23
Birthweight (g)	3711 ± 504
Gestational age at birth (weeks)	39.5 ± 1.37
Time to milk collection post-delivery (days)	15.8 ± 10.58

	N (%)
Milk collected 1–7 days post-delivery	75 (20.6)
Milk collected 7–14 days post-delivery	110 (30.2)
Milk collected 14–30 days post-delivery	151 (41.5)
Milk collected > 30 days post-delivery	28 (7.7)
Delivery mode: C-Section	37 (10.2)
Delivery mode: vacuum extraction	11 (3)
Gestational diabetes	33 (9.1)
Type I diabetes	2 (0.5)
Child gender: male	188 (51.6)
Parous	277 (76.1)
Preeclamptic	6 (1.6)
Smoker during pregnancy	97 (26.6)

### Characterization of human milk EVs

**Figure 1:**
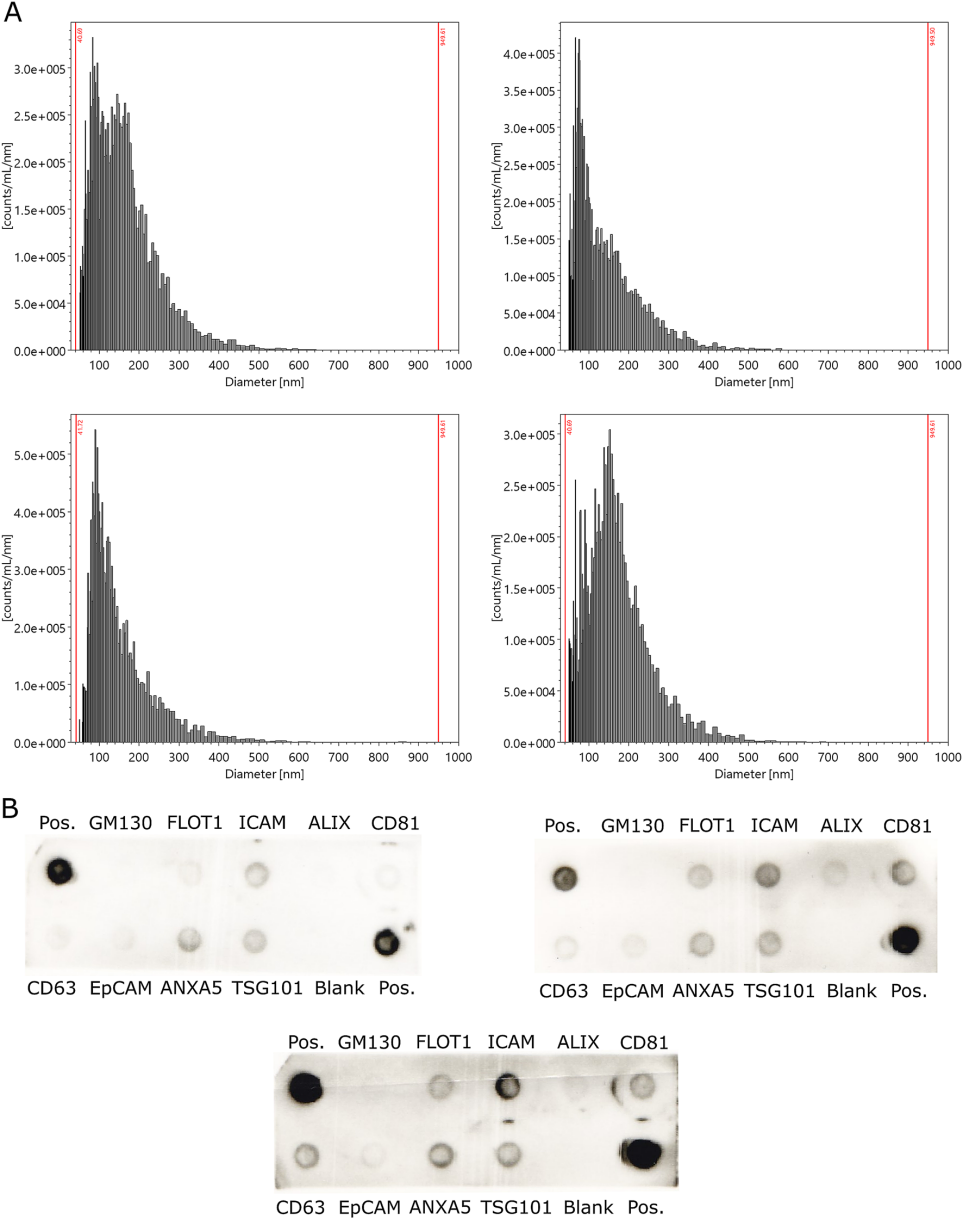
Characteristics of human milk EVs in representative samples. (**A**) Histograms for the number of particles per milliliter of EVs by particle diameter with bins logarithmically scaled. (**B**) Exo-Check Array results for three representative samples demonstrating exosome-related protein expression, where the darkness of the spot indicates the presence of the indicated protein. The absence of a spot for GM130 indicates absence of intracellular contaminants in the EV isolation. Abbreviations: GM130 (Cis-golgi matrix protein), FLOT1: Flotillin-1, ICAM1: Intercellular adhesion molecule 1, ALIX: Programmed cell death 6 interacting protein (PDCD6IP), CD81: Tetraspanin, CD63: Tetraspanin, EpCam: Epithelial cell adhesion molecule; ANXA5: Annexin A5, TSG101: Tumor susceptibility gene 101.

We first characterized EV concentration and size of four random samples using the ViewSizer 3000. The average EV count was 1.5e11 ± 3.4e10 particles/mL skim milk. EV sizes ranged between 50 and 650 nm with the average size 163.5 nm ± 83.5 (Table 2, Fig. 1A). We next characterized three samples for well-known EV protein markers using the Exo-Check Antibody array, which semi-quantitatively measures eight known exosome markers and a marker of cellular contamination (Fig. 1B). We detected our positive control and had no background detection of the blank for all samples. We also detected CD63, CD81, FLOT1, and ICAM in all samples. EpCAM and ALIX were detected faintly in two of the three samples. GM130, the marker for cellular contamination, was detected very faintly in one sample, suggesting that we had little contamination of intracellular vesicles.

**Table 2:** Characteristics of human milk EVs in four representative samples.

Sample ID	Particles/mL skim milk	Average EV size (nm)	Standard deviation (nm)	Coefficient of variation	10th percentile diameter	50th percentile diameter	90th percentile diameter
976	1.22E+11	178	83	0.46	88	161	286
972	1.86E+11	158	84	0.53	84	132	272
335	1.19E+11	150	85	0.57	69	129	265
192	1.73E+11	168	82	0.49	82	153	272
Mean	1.50E+11	163.5	83.5	0.51	80.8	143.8	273.8

### miRNA expression characterization in human milk EVs

We sequenced 364 human milk EV samples using the HTG EdgeSeq miRNA Whole Transcriptome Assay, which targets 2083 mature miRNAs. We detected 447 miRNAs with at least one read in all samples and 1523 miRNAs in at least 70% of samples. Of these 1523 miRNAs, we measured a total of 1.1 billion reads across all 364 samples with an average of 3.1 million reads per library. The top 10 miRNAs made up 76.3% of total reads and on average 75.9% of reads per library. The top four highly expressed miRNAs each represented on average 10% of each total library with reads from miR-4271 composing 15.4% ± 13.4% of reads on average per library (Supplemental Fig. S1A).

Average miRNA expression ranged between 3.20 and 740,567 median ratio normalized counts. When averaged across samples, 565 miRNAs had expression between 0 and 10 counts and 500 had expression between 10 and 50 counts (Supplemental Fig. S1B). We measured 326 miRNAs with greater than 100 average counts, with 114 of those with greater than 1000 average counts, 29 with greater than 10,000 average counts, and seven with greater than 100,000 average counts (Supplemental Fig. S1B).

### Hierarchical clustering of human milk EV miRNAs

miRNAs were clustered using hierarchical clustering, which identified five optimal miRNA clusters (Supplemental Fig. S2). This is an improvement over methods that a priori evaluate the top 10 or 15 expressing miRNAs, as it empirically determines the numbers of miRNAs that can be identified as having “high”, “mid”, and “low” expression. Expression of each miRNA was averaged across samples and cluster to generate summaries by cluster (Table 3), then sorted based on the number of miRNAs per cluster. Larger clusters contained the miRNAs with low expression (12 ± 17 average normalized counts). The highest 15 expressing miRNAs (207,596 ± 269,052 average normalized counts) were grouped into Cluster 1 (miR-4271, miR-3197, miR-2861, miR-6131, miR-6126, miR-6741-5p, miR-6803-5p, miR-6727-5p, miR-149-3p, miR-1237-5p, miR-1207-5p, miR-5739, miR-6088, and miR-8078) (Table 4). The next 27 highest expressing miRNAs were included in Cluster 2. Cluster 3 included 157 miRNAs. To make this category more interpretable, Cluster 3 was divided at the next node and only the next highest expressing 67 miRNAs were included in the pathway analyses. Full summaries from Cluster 1, 2, and 3 are shown in Supplementary Table S1.

**Table 3:** Summary statistics for expression of human milk extracellular vesicle miRNAs in the 5 clusters.

Cluster #	# miRNAs	Mean	SD	Min	Max
1	15	207,596	269,052	6,690	2,480,400
2	27	13,299	21,242	746	195,982
3	157	1208	1050	158	10,058
4	368	100	95	13	1108
5	956	12	17	0	178

*SD* standard deviation, *Min* minimum value, *Max* maximal value.

*Counts are median ratio normalized with DESeq2.

**Table 4:** Summary statistics for expression of top 15 human milk extracellular vesicle miRNAs.

miRNA	Mean counts*	SD	# microT-CDS mRNA targets	# TargetScan mRNA targets	# Tarbase mRNA targets
miR-4271	7.4E+05	9.2E+05	767	950	0
miR-3197	6.0E+05	1.0E+06	26	609	0
miR-2861	4.0E+05	2.2E+05	969	863	277
miR-6131	3.6E+05	1.4E+05	29	136	0
miR-6752-5p	2.8E+05	1.0E+06	21	59	0
miR-6126	2.3E+05	2.6E+05	233	701	0
miR-6741-5p	1.4E+05	1.2E+05	486	373	0
miR-6803-5p	7.1E+04	5.7E+04	271	426	0
miR-6727-5p	7.0E+04	5.2E+04	190	836	0
miR-149-3p	5.8E+04	7.3E+04	428	192	75
miR-1237-5p	4.8E+04	3.9E+04	330	142	0
miR-1207-5p	3.6E+04	3.5E+04	439	33	0
miR-5739	3.4E+04	3.4E+04	41	161	0
miR-6088	3.1E+04	3.7E+04	215	166	0
miR-8078	2.7E+04	1.8E+04	56	67	0

*SD* standard deviation.

*Counts are median ratio normalized with DESeq2.

### Gene ontology and pathway analysis of miRNA clusters

We performed a pathway analysis using the Kyoto Encyclopedia of Genes and Genomes (KEGG)^30–32^ separately for the top three highest expressing miRNA clusters. mRNA targets were predicted using DIANA microT-CDS and TargetScan, and experimentally validated targets were extracted from Tarbase. DIANA MirPATH was used for pathway analysis on each set of targets (Tables 5, 6). Overlapping and significant KEGG pathways between platforms but amongst all clusters were: Fatty acid biosynthesis, adherens junction, focal adhesion, ECM-receptor interaction, signaling pathways regulating pluripotency of stem cells, platelet activation, hippo signaling pathway, endocytosis, Rap1 signaling pathway, thyroid hormone signaling pathway, and PI3K-Akt signaling pathway (Supplemental Table S2) (False discovery rate [FDR] adjusted p-value ≤ 0.05). For Cluster 1, the only overlapping pathways between TargetScan and microT-CDS were focal adhesion and Ras signaling. For cancer-related pathways, proteoglycans in cancer, glioma, endometrial cancer, pathways in cancer, and prostate cancer were identified by all three platforms independent of cluster. Full KEGG pathway results are available in the supplemental information (Supplemental Table S2).

**Table 5:** Top 10 FDR Significant, non-cancer related, KEGG Pathways associated with the human milk extracellular vesicle miRNAs in clusters 1, 2 and 3 determined with DIANA microT-CDS.

KEGG pathway	p-value	# mRNA Targets	# miRNAs	Cluster
**Estrogen signaling pathway**	1.38E−04	29	11	1
Thyroid hormone signaling pathway	1.38E−04	39	12	1
**Axon guidance**	1.11E−03	41	12	1
**ECM-receptor interaction**	1.25E−03	23	12	1
Ras signaling pathway	6.16E−03	66	13	1
Thyroid hormone synthesis	9.29E−03	21	10	1
ErbB signaling pathway	1.16E−02	30	12	1
Focal adhesion	1.31E−02	62	12	1
Insulin secretion	2.23E−02	30	10	1
Gap junction	2.23E−02	26	12	1
Fatty acid biosynthesis	3.69E−05	3	2	2
Endocytosis	4.95E−04	90	22	2
Glycosphingolipid biosynthesis—lacto and neolacto series	2.65E−03	9	9	2
Signaling pathways regulating pluripotency of stem cells	2.65E−03	57	19	2
Adrenergic signaling in cardiomyocytes	2.65E−03	62	21	2
**ECM-receptor interaction**	2.66E−03	35	17	2
FoxO signaling pathway	3.72E−03	55	18	2
**Estrogen signaling pathway**	3.73E−03	41	19	2
**Axon guidance**	4.63E−03	57	19	2
Regulation of actin cytoskeleton	4.63E−03	87	22	2
Mucin type O-Glycan biosynthesis	3.58E−09	23	33	3
Morphine addiction	1.04E−08	71	52	3
GABAergic synapse	1.04E−07	66	50	3
Focal adhesion	2.03E−07	158	58	3
**Axon guidance**	2.06E−06	98	56	3
Circadian entrainment	2.63E−05	73	53	3
Adherens junction	2.71E−05	57	51	3
ErbB signaling pathway	3.95E−05	69	50	3
**ECM-receptor interaction**	4.96E−05	60	45	3
**Estrogen signaling pathway**	1.44E−04	73	55	3

*KEGG* Kyoto Encyclopedia for genes and genomes, *FDR* false discovery rates.

Bold: Pathways shared between all clusters.

**Table 6:** Top 10 FDR Significant, cancer related, KEGG Pathways associated with the human milk extracellular vesicle miRNAs in clusters 1, 2 and 3 determined with microT-CDS.

KEGG pathway	p-value	# mRNA Targets	# miRNAs	Cluster
**Glioma**	2.88E−03	23	10	1
Melanoma	4.15E−02	23	10	1
Proteoglycans in cancer	2.46E−07	83	21	2
Pancreatic cancer	2.65E−03	32	15	2
**Glioma**	2.65E−03	30	16	2
Choline metabolism in cancer	2.65E−03	48	18	2
Renal cell carcinoma	8.26E−03	30	16	2
Non-small cell lung cancer	1.76E−02	26	13	2
Prostate cancer	1.99E−02	41	17	2
Endometrial cancer	2.63E−02	26	15	2
Colorectal cancer	3.20E−02	29	17	2
Proteoglycans in cancer	4.21E−09	142	57	3
**Glioma**	1.04E−05	51	52	3
Pathways in cancer	1.04E−05	275	66	3
Pancreatic cancer	7.81E−04	50	53	3
Prostate cancer	7.81E−04	67	57	3
Non-small cell lung cancer	8.71E−04	42	50	3
Endometrial cancer	1.18E−03	41	50	3
Renal cell carcinoma	2.42E−03	50	48	3
Melanoma	3.27E−03	53	55	3
Small cell lung cancer	1.00E−02	62	47	3

*KEGG* Kyoto Encyclopedia for genes and genomes, *FDR* false discovery rates.

Bold: Pathways shared between all clusters.

Gene Ontology (GO) category analysis was conducted on mRNA targets from Cluster 1 (Table 7). MicroT-CDS and TargetScan predicted 95 and 92 GO categories respectively, with 75 overlapping categories. From the top 10 GO categories seven overlapped between TargetScan and microT-CDS: organelle, cellular nitrogen compound metabolic process, biosynthetic process, symbiosis encompassing mutualism through parasitism, ion binding, small molecule metabolic process, and neurotrophin TRK receptor signaling pathway (Supplemental Table S3). Heatmaps of miRNA GO categories demonstrate that even though all 15 miRNAs were included in the top GO categories, a subset of seven miRNAs (miR-6088, miR-6752, miR-149, miR-1207, miR-6131, miR-4271, and miR-6126) were driving the categorization (Supplemental Fig. S4). Full GO results are available in Supplemental Table S3.

**Table 7:** Top 10 FDR Significant, GO categories associated with the high expressing 15 human milk extracellular vesicle miRNAs in cluster 1, determined with microT-CDS.

GO category	p-value	# mRNA Targets	# miRNAs
Organelle	6.80E−106	1965	15
Ion binding	2.86E−55	1207	15
Cellular nitrogen compound metabolic process	5.47E−51	952	15
Biosynthetic process	1.26E−41	826	15
Nucleic acid binding transcription factor activity	8.58E−23	244	15
Cellular protein modification process	1.46E−19	458	15
Gene expression	1.49E−19	135	14
Small molecule metabolic process	9.28E−19	456	14
Symbiosis, encompassing mutualism through parasitism	3.39E−17	125	14
Neurotrophin TRK receptor signaling pathway	9.07E−17	70	12

*GO* gene ontology, *FDR* false discovery rates.

### Correlations between miRNAs: top expressed miRNAs from clusters 1–3

**Figure 2:**
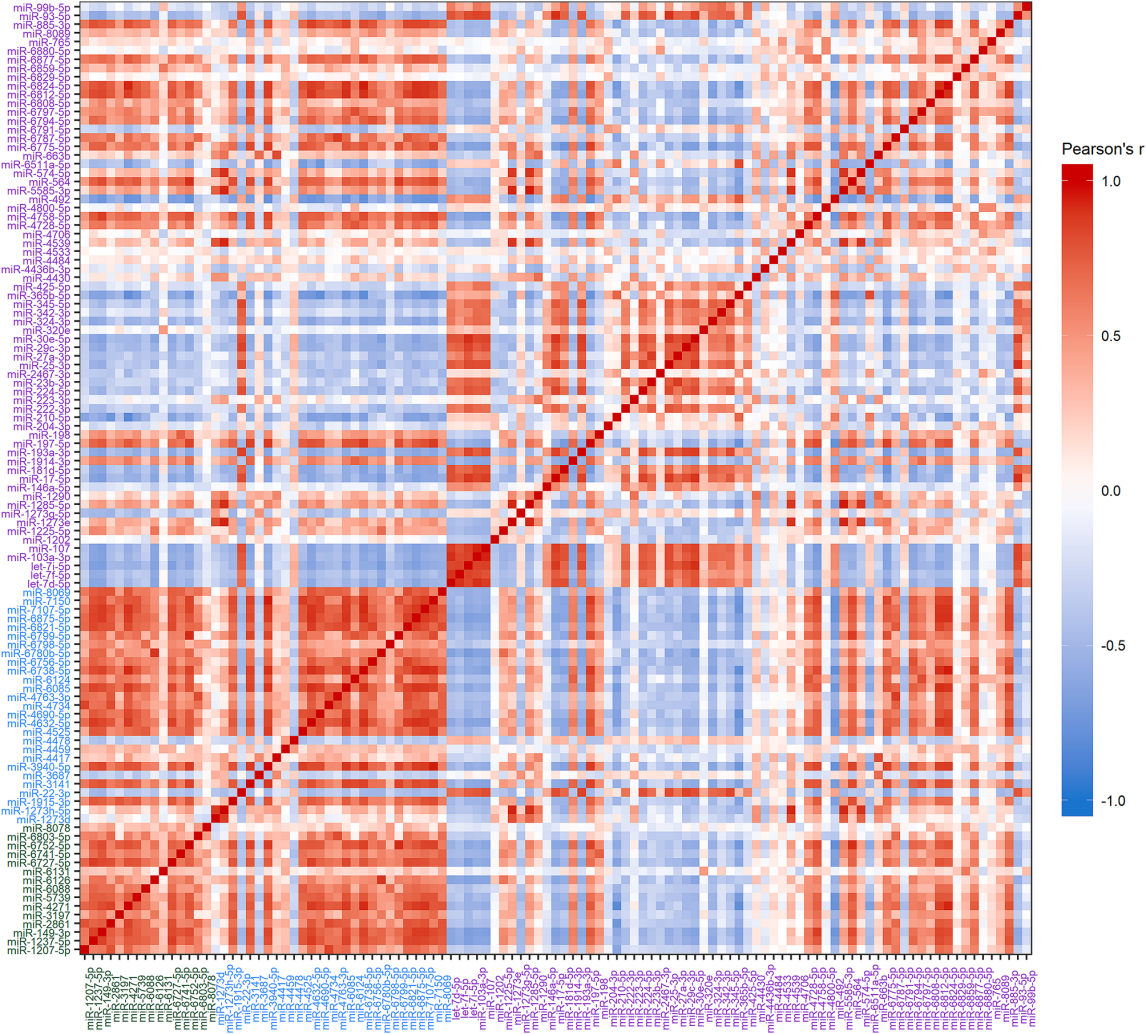
Pearson’s correlations between the 109 miRNAs in the top 3 clusters sorted by expression cluster membership. Gradient indicates the Pearson’s *r* with red indicating positive correlations and blue negative correlations. Color of label indicates original cluster membership (Green: Cluster 1; Blue: Cluster 2; Purple: Cluster 3).

**Figure 3:**
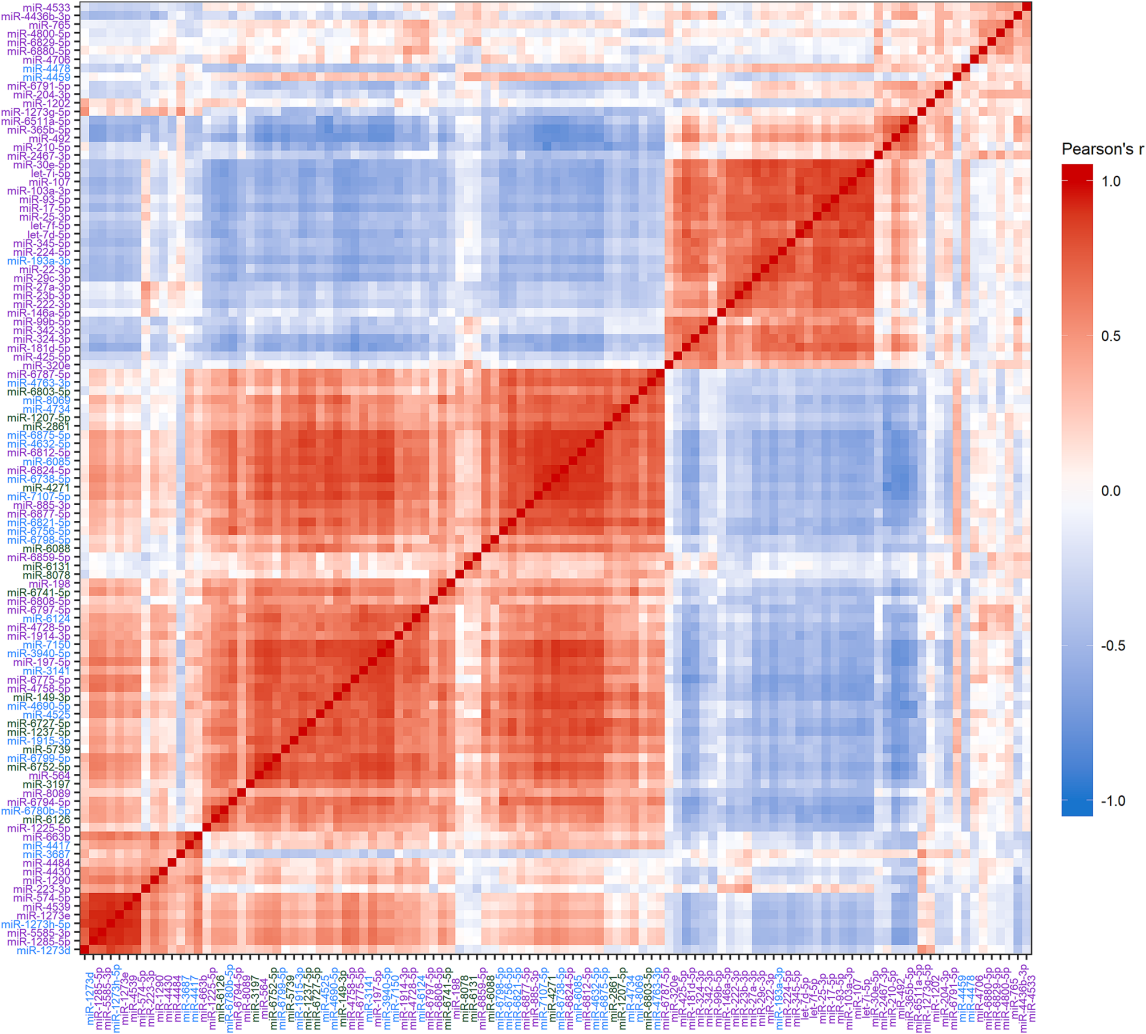
Pearson’s correlations between the 109 miRNAs in the top 3 clusters then re-clustered by correlation coefficient using hierarchical clustering with Euclidian distance and the complete linkage method. Gradient indicates the Pearson’s *r* with red indicating positive correlations and blue negative correlations. Color of label indicates original cluster membership (Green: Cluster 1; Blue: Cluster 2; Purple: Cluster 3).

Pearson’s correlation plots of the 109 miRNAs within the top 3 clusters show that although expression of the top 15 Cluster 1 miRNAs (Green labels) are generally correlated, the correlations between the 67 miRNAs in Cluster 3 are not consistently correlated (Purple labels) (Fig. 2). Clustering of miRNAs by correlation demonstrates that expression patterns do not mirror correlation patterns between miRNAs (Fig. 3). High positive correlations (Pearson’s *r* ≥ 0.7) were detected between 588 pairs of miRNAs, and some miRNAs were highly correlated with many other miRNAs. For instance, miR-6812-5p was highly positively correlated with 29 different miRNAs and miR-6824-5p was highly correlated with 28 miRNAs. The strongest positive correlations were measured between miR-5585-3p and miR-1285-5p (*r* = 0.97) and between miR-7150 and miR-3940-5p (*r* = 0.96). High negative correlations (*r* ≤  − 0.7) were detected between 38 pairs of miRNAs. miR-492 was inversely correlated with 15 miRNAs and miR-365b-5p was inversely correlated with 11 miRNAs. The strongest negative correlations measured were between miR-210-5p and miR-4271.

### Associations of human milk miRNAs with milk collection day post delivery

**Figure 4:**
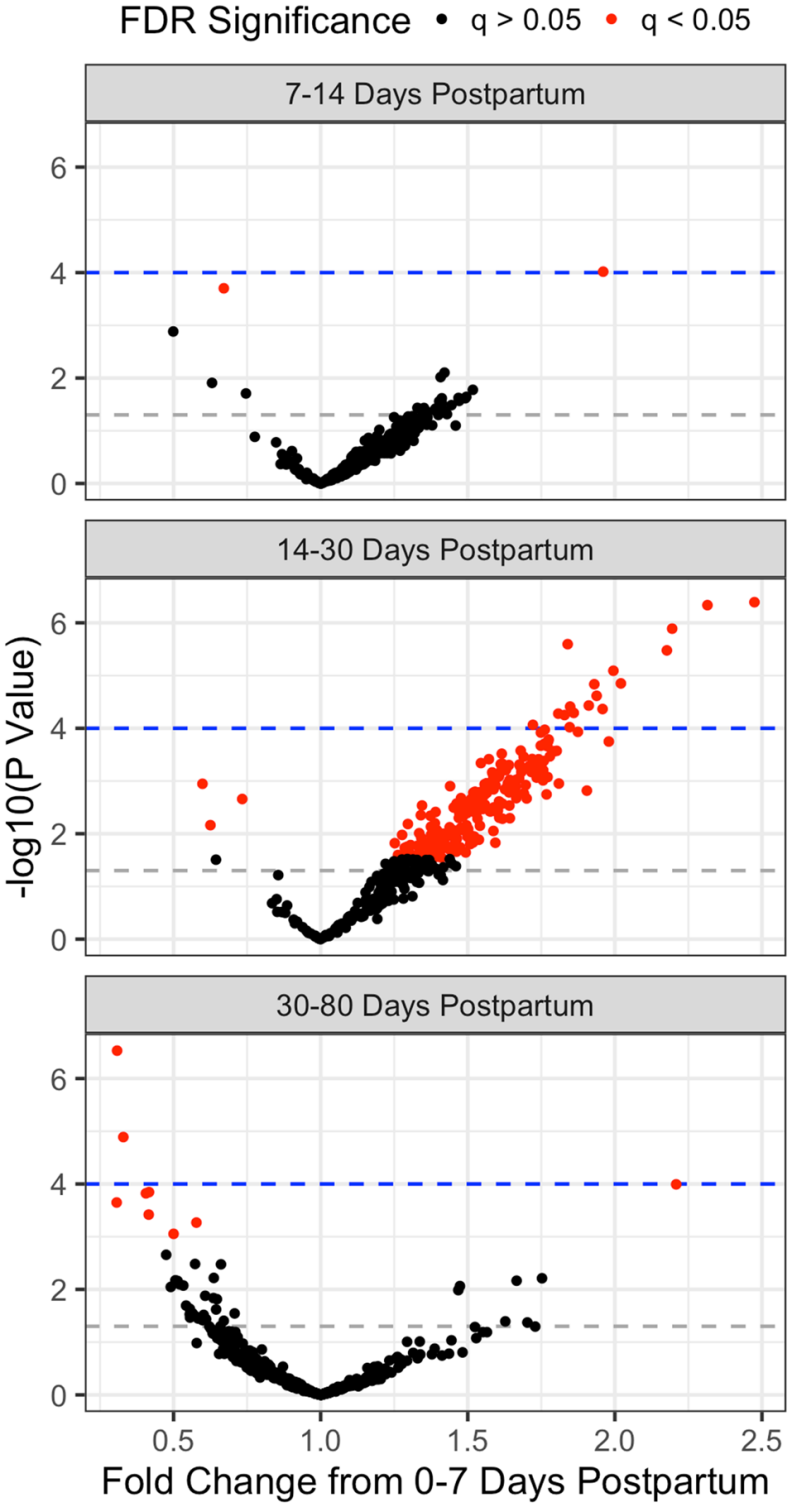
Volcano plots for associations between human milk EV miRNA and timing of milk collection post-delivery, where each point represents an effect estimate as fold change (x-axis) and − log10(p-value) (y-axis) for an individual miRNA with 0–7 days post-delivery as the reference category. Red points are significant after correction for false discovery rate (FDR). Gray dashed line represents p = 0.05 and blue dashed line represents Bonferroni significance (p = 0.0001). All models adjusted for: maternal age, BMI, smoking, parity and RNA starting concentration.

Milk was collected between 1 and 80 days post-delivery, and we divided this in to four categories for analysis: 0–7 days post-delivery, 7–14 days, 14–30 days, and 30–80 days (Table 1), with 0–7 days serving as the reference category. Following correction for multiple testing, only two miRNAs were significant different between 0–7 and 7–14 days post-delivery at q ≤ 0.05 (Fig. 4, top panel). miR-128-3p was upregulated during this period (Fold change with 95% confidence interval: 1.96 (1.40, 2.75), q = 0.04), while miR-6799-5p was down regulated (Fold change 0.67 (0.54, 0.83), q = 0.04). In contrast, 246 out of 419 miRNAs were significantly altered from 14 to 30 days post-delivery. The expression of the majority of these miRNAs were upregulated during this period (243/246) and three miRNAs were down regulated. The top four most significantly upregulated miRNAs were miR-152-3p, miR-511-3p, miR-345 and miR-4534 (Fold changes 2.31 (1.67, 3.21), 2.47 (1.74, 3.51), 2.19 (1.60, 3.02), and 1.84 (1.43, 2.37), respectively, q < 10^–4^ for all). The down regulated miRNAs were miR-1290 (0.60 (0.44, 0.82)), miR-27a-5p (0.63 (0.45, 0.88)), and miR-6799-5p (0.733 (0.60, 0.89)). Finally, expression of 8 miRNAs (miR-1290, miR-130a-3p, miR-146a-5p, miR-195-5p, miR-27b-3p, miR-34a-5p, miR-612, miR-6799-5p) decreased in 30–80 days post-delivery in comparison to the first week, and expression of one miRNA increased (miR-6780b-5p) (Fig. 4, bottom panel). Full results for associations between milk collection date and EV miRNAs can be found in Supplemental Table S5.

### Associations of human milk EV miRNAs with maternal characteristics

**Figure 5:**
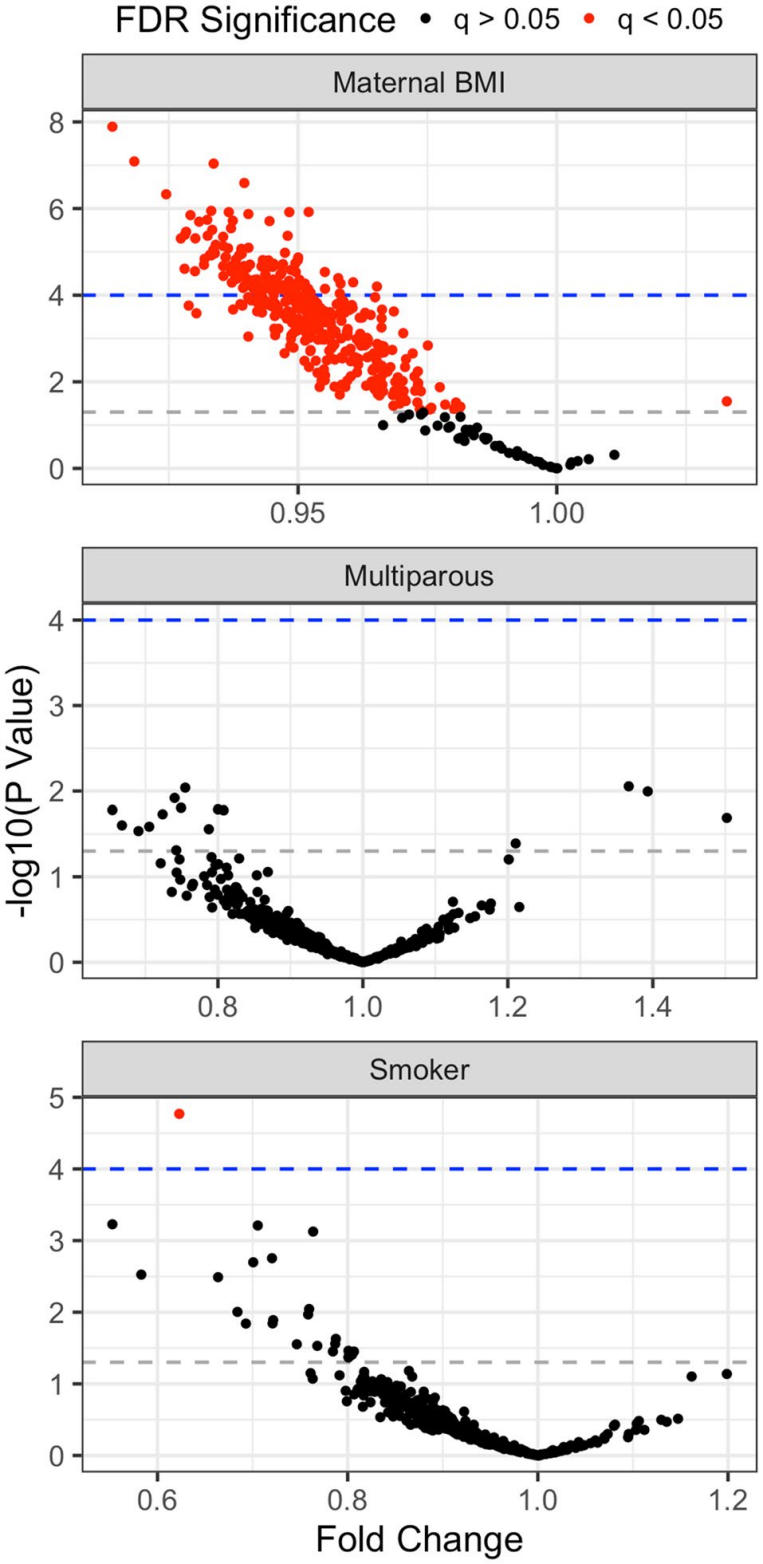
Volcano plots for associations between human milk EV miRNA and maternal characteristics where each point represents an effect estimate as fold change (x-axis) and − log10(p-value) (y-axis) for an individual miRNA. Effect estimates for maternal BMI (top panel) represent fold change per 1 kg/m^2^ increase in BMI, for parity (middle panel) represent fold change from nulliparous to multiparous, and for smoking represent fold change from nonsmoker to smoker. Red points are significant after correction for false discovery rate (FDR). Gray dashed line represents p = 0.05 and blue dashed line represents Bonferroni significance (p = 0.0001). All models adjusted for: collection period post-delivery, maternal age, BMI, smoking, parity, RNA starting concentration.

We next evaluated milk EV miRNA expression with maternal BMI (in kg/m^2^), smoking status (smoked during pregnancy or not), and parity (nulliparous or multiparous). Of 419 miRNAs evaluated, 374 were negatively associated with BMI (Fig. 5, top panel). miR-4769-5p was weakly, but significantly, positively associated with BMI (Fold change 1.03 (1.00, 1.06) per kg/m^2^ increase in BMI, q = 0.03). The top four miRNAs most significantly negatively associated with BMI were miR-128-3p (miR-130a-3p, miR-574-3p, and miR-6881-5p (Fold changes 0.91 (0.89, 0.94)), 0.93 (0.91, 0.96), 0.92 (0.89, 0.95), and 0.94 (0.92, 0.962), respectively). There were no miRNAs significantly associated with parity (Fig. 5, middle panel) and only one miRNA was significantly negatively associated with maternal smoking after FDR correction (miR-6780b-5p, fold change 0.62 (0.50, 0.77)) (Fig. 5, bottom panel). Full results can be found in Supplemental Table S6.

## Discussion

Human milk transfers important nutrients and biological signals from the mother to the infant. Many of these signals are nucleic acids encapsulated within EVs. Here we presented the largest study to date on human milk EV-miRNAs. We have found that most of human milk EV-miRNAs are not highly expressed, but many EV-miRNAs can be also detected at moderate, or even at high, levels. We also identified that human milk EV-miRNAs may play a role in endocrine signaling, cellular community, and neurodevelopment. We have further shown that many miRNAs are associated with milk collection period post-delivery and maternal BMI.

In this study of 364 human milk EV samples, we detected 1,523 unique miRNAs with at least one read in 70% of samples. Our study outnumbers previous human milk EV-miRNA studies, which have reported 602 miRNAs^13^, 898 miRNAs^7^, and 221 miRNAs^29^ in samples collected between 24 h and 10 months postpartum. Previous research has also suggested that the majority of sequencing reads in human milk miRNA samples are dominated by a few miRNAs^7,12,13,29^, which was the case in our analysis. The top 15 highly expressed miRNAs identified in the present study are distinct from those in previous research^13,22,25–27,29,33^. However, we detected nearly all highly expressed miRNAs in previous studies within our top 3 clusters. For instance, miR-148-3p was identified as one of the most highly expressed miRNAs in four previous studies^12,13,27,33^, and we identified it as part of Cluster 3. These discrepancies may be due to differences in technical methods or to population differences between studies. For instance, the population of the Faroe Islands trace ancestry back to Scandinavia and the British Isles^34^, which may contribute to these differences. Additionally, the HTG EdgeSeq library preparation method uses a distinct set of mature miRNA probes, which eliminates the need for reverse transcription, adenylation or adapter ligation in library preparation, and may produce a differences in library preparation bias^35–37^. Other discrepancies may arise from differences in EV isolation methods. We used a membrane-affinity based capture of EVs for the current analysis and included both exosomes and microvesicles. However, other previous studies have focused exclusively on exosomes derived from ultracentrifugation^29,38^ or from precipitation-based^7,13^ methods. Membrane-affinity columns and ultracentrifugation are both recognized as having intermediate recovery and specificity by the International Society for Extracellular Vesicles in comparison to precipitation-based kits which have high yield but low specificity^39^. Thus, differences in preparation methods or miRNAs present in contaminating protein complexes may lead to differences in miRNAs detected.

Of the 15 human milk EV-miRNAs that cluster by the highest average level of expression across the population, many have endocrine-related mRNA targets (e.g. thyroid hormone signaling and synthesis, estrogen signaling, and insulin secretion) and signal transduction targets (ErbB and Ras Signaling). These pathways are highly relevant for mammary gland development and lactation. Mammary gland development is regulated by reproductive hormones estrogen and progesterone, which increase throughout pregnancy leading to a decrease in breast adipose tissue, expanded ductal tissue, and increased lobular branching^40,41^. Stimulation of the pituitary gland then increases prolactin production in preparation for milk production and immediately after birth estrogen and progesterone decrease to allow prolactin release to induce lactation. In turn, thyroid hormones may regulate estrogen receptor beta and prolactin signaling during pregnancy and early postpartum in vivo^42^. Insulin receptor signaling is also required for mammary differentiation during pregnancy in animal models^43^ and stimulates milk protein production in vitro^44,45^. Furthermore, the ErbB family of type I receptor kinases are involved in ductal and alveolar morphogenesis during pregnancy^46^. Hence, the pathways identified by miRNA expression in this population are indicative of essential mammary gland development and milk production processes.

Across all three human milk EV-miRNAs clusters, a number of pathways were identified within the category of cellular community and signaling interactions. These pathways may be related to the role of EVs in intercellular communication. Other pathways were related to the nervous system function and programming, which, given the critical role for breastfeeding in neurodevelopment^47,48^, may represent important biological signals for infants. We also identified several pathways related to various cancers, particularly glioma. Many of these pathways were also reported by previous studies on the lipid fraction of human milk^25,33^. Other studies have suggested the immune system as a key target of human milk miRNAs^13,24,33^, which is significant as breastfeeding plays a key role in establishment of the infant immunity. However, with a couple of exceptions (platelet activation and symbiosis, encompassing mutualism through parasitism), we did not observe a strong enrichment for the immune system in our EV-miRNA samples. Human milk immunomodulation and resistance to infections are related to non-miRNAs factors, such as immunoglobulins. However, this discrepancy may be related to several other factors as discussed above, including timing of milk collection.

GO analysis established several general biological, cellular, and molecular processes critical for cell function, with organelle being the most strongly enriched cellular component. Ion binding was the most significantly enriched molecular process, which is unsurprising as calcium is a key nutrient in human milk for the infant and calcium signaling is important for lactation^49^. Several metabolic processes were also identified, including cellular nitrogen compound metabolic process, which was the most enriched biological process, and was also identified in a previous study examining preterm human milk^25^.

The highest expressed miRNAs in our study population were miR-4271, miR-3197, and miR-2861, respectively. Though little information on miR-4271 is available, one study found miR-4271 to be expressed in multiple human tissues and at high levels in the liver and intestines^50^. Interestingly, a variant in the apolipoprotein C-III (*APOC3*) gene was found to create a miR-4271 binding site in the corresponding mRNA, which was associated with lower plasma triglycerides, potentially increasing risk of coronary heart disease^50^. Other studies demonstrated that miR-4271 expression was upregulated in the plasma of smokers in comparison to nonsmokers^51^ and following hypoxia in human embryonic stem cells^52^, suggesting that miR-4271 may be responsive to environmental stressors though its function in human milk remains unknown. The second most highly expressed miRNA in human milk EVs, miR-3197, has been suggested to be semen-specific in forensic studies^53,54^, however, miR-3197 expression was detected in human mesenchymal stem cells^55^, human placenta^56^, serum from patients with hepatocellular carcinoma^57^. miR-1286 is a putative HDAC5 (histone deacetylase 5) repressor, which in turn regulates RUNX2 (Runt-Related Transcription Factor 2), and playing a role in osteogenesis and osteoporosis^58–60^.

We examined correlations between highly expressed miRNAs in our population to determine which miRNAs may be co-regulated. Overall, miRNAs were highly correlated, with many more positive correlations between miRNAs detected than negative correlations. Those with the highest correlations were not located on the same chromosomes, suggesting possible trans-regulation or shared response elements or transcription factor regulation. miR-6812-5p and miR-6824-5p were each highly correlated with many other human milk EV-miRNAs. However, little experimental research is available on their functions. miR-6824-5p was upregulated in osteoporosis^61^ and downregulated in osteoarthritis^62^ in two independent studies. A highly positive correlation was detected between miR-5585-3p and miR-1285-5p. In a study on enteroviral infections, miR-5585-3p and miR-1285p were two of six miRNAs altered in cells following infection on two types of viruses^63^. miR-492 was inversely correlated with 15 miRNAs, and is hypothesized to play a key role in several cancers, including breast cancers^64–66^. miR-365b-5p was inversely correlated with 11 miRNAs. Two experimentally validated targets of miR-365b-5p are collagen genes, suggesting a role for miR-365b-5p in skeletal formation, with several studies implicating miR-365b-5p in chondrogenesis^67,68^.

Next, we examined associations between miRNAs and milk collection period, finding many miRNAs differed between the first week and days 14–30 post-delivery, suggesting large changes in miRNA production or secretion during this period. Previous studies investigating the effect of time post-delivery on human milk miRNAs, have also found variation in miRNA expression over time. For instance, a study investigating miR-30b-5p, miR-let7a-5p, and miR-378 levels in total colostrum vs mature milk found decreased levels of let-7a and miR-378a-5p^23^, which we also observe between 0–7 and 14–30 days. A study investigating changes in human milk lipid miRNAs at 2 months, 4 months and 6 months postpartum, found upregulation of over 200 miRNAs in month 4. While we also observed large alterations in miRNAs with time, this work cannot be directly compared to our own, owing to differences in milk collection time post-delivery and large differences between miRNAs in human skim milk and lipids^22^.

Many of the miRNAs altered between 14 and 30 days have been found in previous studies to have roles related to mammary gland development. For instance, miR-128-3p was upregulated in the second week postpartum and is known to target RAC1 (Rac family small GTPase 1), the key GTPase in clearance of apoptotic cells during involution in mammary gland remodeling^69,70^. miR-27a-5p was downregulated in milk EVs. Previous research demonstrated that miR-27a-5p expression increased in the mammary glands of rats between one and seven days postpartum^71^ and that it’s expression in the goat and bovine mammary gland was correlated with prolactin expression and regulated milk triglyceride content^72,73^. Other studies have found that miR-345, which was upregulated in days 14–30 post-delivery in the present analysis, may have a role as a tumor suppressor and can inhibit adipocyte differentiation via targeting of VEGF-B (vascular endothelial growth factor B) in vitro^74^.

Additionally, a number of miRNAs were downregulated between 30 and 80 days after delivery. The most strongly downregulated miRNA, miR-146a-5p, plays a critical role in regulation of the innate immune inflammatory response^75^. Another miRNA, miR-130a-3p, may regulate prolactin, which is critical to mammary gland development and milk production. miR-130-3p overexpression decreased both protein and RNA prolactin levels in GH3 pituitary cancer cells, possibly through targeting ERα^76^. Furthermore, several of these miRNAs (miR-130a-3p, miR-27b-3p, and miR-34a-5p) have been validated to target PPARγ (peroxisome proliferator-activated receptor γ) in vitro^77,78^. PPARγ, a nuclear receptor found primarily in adipose tissue, is a well-characterized regulator of mammalian adipogenesis and a key driver of expression of genes that result in lipid and triglyceride accumulation. Previous studies in vivo found that lactating dams with endothelial cell specific PPARγ knockdown produced milk containing high levels of inflammatory lipids to offspring, resulting in growth retardation and alopecia^79^. A study on changes in milk fatty acid content in lactating women found that PPARγ levels in milk decreased up to 30-fold beginning at four days post-delivery^80^. A study in bovine mammary epithelial cells found that miR-130a-5p over expression resulted in decreased PPARγ mRNA and protein, and decreased triacylglycerol synthesis^81^.

Interestingly, miR-130a-5p was also strongly negatively associated with maternal pre-pregnancy BMI, suggesting a role in adipogenesis or milk fat synthesis. The miRNA most negatively associated with BMI was miR-128-3p, which also targets PPARγ in mice^82^ and can inhibit adipogenesis in vitro^83^. In the present study, miR-129b-3p expression decreased with increasing BMI, however, a previous study examining EVs isolated from 1st trimester plasma found miR-29b-3p levels greater in women with gestational diabetes than in controls^84^. A previous study investigated associations between maternal weight characteristics and three miRNAs involved in adipogenesis, miR-30b-5p, miR-let7a-5p, and miR-378, measured in total colostrum from 86 mothers^23^. Researchers found these miRNAs negatively correlated with maternal pre-pregnancy BMI. Similarly, we found negative associations between these miRNAs and maternal pre-pregnancy BMI in the present study. In particular, miR-30b-5p was the 19th most significantly negatively associated miRNA with BMI.

Finally, we also investigated associations between miRNAs and maternal parity and smoking. Mammary gland physiology changes greatly after the first pregnancy^85^, and we hypothesized that these differences might impact miRNA abundance in milk. However, we observed no miRNAs significantly associated with parity in our population. Additionally, a relatively high percentage of women in our study population smoked during pregnancy. Studies have suggested that maternal smoking alters EV-miRNA profiles in maternal plasma^86^. However, after correction for false discovery, only one miRNA remained significantly associated with maternal smoking. Unfortunately, little information is available on miR-6780b-5p and more research is needed to understand the functional significance of this miRNA.

Our study has some limitations. Firstly, as human milk samples were frozen prior to removal of cellular material, we likely had some contamination by intracellular vesicles from lysed cells. However, our representative samples showed minimal contamination by GM130, a cis-Golgi matrix protein that can be used as a marker of cellular contamination. Additionally, our samples were stored for many years before analysis and we are unable to characterize the effects of long-term storage on our samples. Furthermore, we were unable to characterize the milk EVs within our samples using electron microscopy, which could provide confirmation that our preparation of EVs is pure and provide insight into the subpopulations of EVs present. However, we utilized a well-accepted method of EV isolation and were able to characterize the EV size, distribution and eight common exosome membrane protein markers in representative samples. Additionally, although the HTG Whole Transcriptome miRNA assay profiles over 2000 mature miRNAs, it does not allow for the discovery of novel miRNAs, iso-miRNAs, or miRNA precursors, which may be present in our samples. Nonetheless, the HTG sequencing method is fully automated, making it simple to perform and reproducible, and removes certain biases present in other library prep methods. Limitations of our populations included the lack of information regarding the influence of medications on miRNAs in human milk, such as antibiotic treatments, as well as whether women were actively breastfeeding or weaning at the time of milk collection. Strengths of this study include the fairly large sample size compared to previous studies, the novel library preparation method, and the advanced statistical analysis.

In conclusion, we expand on previous research on human milk EV-miRNA expression and potential function by profiling 364 human milk EV samples from a population of women from the Faroe Islands. We establish that the majority of human milk EVs-miRNAs show low expressions levels; however, several hundred EV-miRNAs can be detected at moderate levels in a fairly large sample size. We further determined potential functions of these miRNAs using pathway analysis, and identified a potential role in endocrine signaling, cellular community, and neurodevelopment as key pathways. Finally, we demonstrate that miRNA expression is associated with timing of milk collection after delivery and maternal BMI.

## Methods

### Study population and milk collection

We studied human milk from women from the Faroe Islands recruited in a population-based prospective cohort study at the time of their pregnancy between 1997 and 2000^87,88^. The cohort initially enrolled 656 mother–child pairs at 34 weeks of gestation at the National Hospital in Tórshavn, Faroe Islands. Only singleton births were included. Included births represent approximately 60% of all pregnancies in Tórshavn during the study period. Most of the attrition was due to work schedules in the busy ward or scheduling conflicts. The Faroese are a North Atlantic fishing community which is fairly homogeneous in regard to socioeconomic and lifestyle factors. Information about maternal age at delivery, parity, pre-pregnancy BMI, gestational weight gain, smoking during pregnancy, gestational diabetic status, preeclampsia, gestational age and child sex was extracted from the obstetric records. Offspring weight (nearest 0.1 kg) was measured by the midwife at milk collection.

Milk was collected at clinical follow-up visit that was planned for two weeks after the expected delivery date (as measured by ultrasound) and which occurred between 2 and 74 days post-birth. Milk was collected either by hand expression or by pump from either breast. Due to the early time of collection and cultural norms, mostly women were likely exclusively breastfeeding at this time. Information on whether milk was expressed before or after feeding was unavailable, however, previous studies have shown that there are little differences in miRNAs between foremilk and hindmilk^25^. Milk was frozen at − 80 °C until analysis. Out of 656 initially enrolled, 364 mothers (55%) had available milk samples and were included in this analysis. All protocols were approved by the institutional review boards of Harvard T.H. Chan School of Public Health, Columbia University and the Scientific Ethical Committee of the Faroe Islands. Written informed consent was obtained from all women. All methods were carried out in accordance with relevant guidelines and regulations.

### Human milk extracellular vesicle isolation

Stored human milk samples (1–2 mL) were thawed on ice, centrifuged at 200×*g* at 4 °C for 10 min to remove the lipid layer, then 3000×*g* at 4 °C for 15 min to remove cellular debris and apoptotic bodies. EVs were extracted with the ExoEasy Maxi kit (Qiagen), which uses the principle of membrane-affinity purification to isolate EVs, according to the manufacturer’s instructions into 400 μL elution buffer, and 20 μL was separated for verification.

### Extracellular vesicle characterization

To confirm EV concentrations, sizes, and distributions, four random human milk EV samples were analyzed using nanoparticle tracking analysis on the ViewSizer 3000 (Horiba Scientific), which uses the principles of Brownian motion with three different lasers and a color camera to detect light scattering of differently sized particles. This method is ideal for polydisperse samples, such as EVs. Samples were diluted from 1:5000 to 1:7000 in sterile PBS for an optimal number of particles per video. The camera was set at a frame rate of 30 frames per second for a 15 ms exposure period and at a gain of 30. We recorded 60, 30 s videos with 300 frames per video. The blue, green and red lasers were set at 210 mW, 12 mW and 8 mW, respectively. Samples were normalized to a blank PBS sample.

To confirm the presence and purity of EVs, we measured levels of eight common EV markers and a cellular control using the Exo-Check Exosome Antibody Array (System Biosciences) according to the manufacturer’s instructions. Protein was quantified with the BCA assay and blots were imaged with the Azure 400 Visible Fluorescent Western Blot Imaging System (Azure Biosystems). We have submitted all relevant data of our EV experiments to the EV-TRACK knowledgebase (EV-TRACK ID: EV200197)^89^.

### Human milk EV-RNA isolation

Human milk EV-RNAs were isolated from the remaining EV fraction with the miRNeasy Serum/Plasma Maxi kit using a 1:5 ratio of sample to Qiazol (Qiagen, Germantown, MD), then cleaned using the RNA Clean & Concentrator-5 Kit (Zymo Research, Irvine, CA). RNA quantity and quality was checked on a Implen NanoPhotometer spectrophotometer (München, Germany) and verified with the TapeStation 4200 total RNA ScreenTape chips (Agilent, Santa Clara, CA) prior to sequencing. As EV RNA is primarily small RNA with no intact ribosomal RNA present, we thus qualitatively examined our samples TapeStation traces. These traces were compared to other traces of milk EV miRNAs and were comparable to previous findings (data not shown).

### miRNA sequencing and processing

HTG EdgeSeq technology (HTG Molecular Diagnostics, Inc., Tucson, AZ) was used to sequence 2,083 mature miRNAs from human milk EV-RNA samples (30 ng input). HTG EdgeSeq is a novel sequencing based platform that uses a probe-based library preparation method, the EdgeSeq miRNA Whole Transcriptome Assay, followed by Illumina HiSeq 4000 sequencing to profile a large number of miRNAs with great sensitivity and specificity^90^. This method provides advantages over traditional library preparation methods^36,37^, as it does not require reverse transcription, adenylation, or adapter ligation, which can induce bias in sequencing results^90^. Samples were barcoded during library prep and multiplexed for sequencing so that 90 samples, three duplicate human milk EV RNA internal controls, and three internal human brain tissue controls were sequenced per run, with four sequencing runs in total. All library preparation and sequencing was performed at the University of North Carolina-Chapel Hill High Throughput Sequencing Facility and data alignment and preprocessing at HTG Molecular Diagnostics in Tucson, AZ. Illumina CASAVA was used for base calling and generation of FASQ files. HTG EdgeSeq Parser software (version 10196100G; https://www.htgmolecular.com/) was used for alignment to a priori defined target sequences. Quality requirements include percentage of bases with a quality score great than 30 (Q30 score) ≥ 85%, percentage of clusters passing filter ≥ 75%, and 180 < CD < 290. All samples passed HTG quality control.

Only miRNAs with at least one read count in 70% of samples were included in the analysis. For miRNA expression profiling, sequencing reads were normalized within and across plates using the median ratio normalization method^91^ in the DESeq2 package version 1.30.0 for R (https://bioconductor.org/packages/release/bioc/html/DESeq2.html). For hierarchical clustering and correlation analysis, a constant was added to avoid zero values and normalized reads were log2 transformed to approximate normal distributions. Values were then corrected for technical batch effects with ComBat^92^. Correlations between four human milk EV triplicate samples ranged between 0.87 and 0.95 across the four sequencing runs and known internal brain control replicate sample correlations ranged between 0.94 and 0.97, demonstrating high reproducibility between samples.

### miRNA statistical analysis

Unsupervised hierarchical clustering was used to empirically determine groups of miRNAs based on expression levels, with Euclidian distance to calculate the distance between observations, and the complete linkage method to compute the clusters. The optimal number of clusters was confirmed with K-means clustering (Supplemental Fig. S4). Pearson’s correlation coefficients were computed between miRNAs in the top three highest expressing clusters, and hierarchical clustering was repeated on the correlation matrix.

Associations between miRNAs, collection time (0–7 days, 7–14 days, 14–30 days, and 30–80 days), maternal BMI, parity (nulliparous/multiparous), and maternal smoking (nonsmoker/smoker) were assessed for miRNAs with greater than 4 log2CPM expression, which was the point of strong agreement between technical replicates, resulting in 419 miRNAs for analysis. One participant with a library with low read counts was removed (final N = 363). We assessed the associations with on raw miRNA counts using generalized linear models with a negative binomial link function to avoid model misspecification due to overdispersion. miRNA counts were first corrected for batch using Combat_Seq^93^. Library normalization factors were calculated as above with DESeq2 and applied to library sizes, which were then included as the offset. Each miRNA was modeled individually, and all maternal factors were considered together in a single model co-adjusted for maternal age and the natural log of RNA starting concentration. To avoid undue influence by large miRNA outliers but ensure that we maintained true biological variation, for each miRNA model, individuals with studentized residuals > 5 were excluded from the analysis. This resulted in removal a total of 12 miRNAs from 8 individuals, leading to a sample size of 362 for 11 out of 419 miRNAs and 361 for 1 miRNA. Model coefficients were exponentiated to the relative rate, which represents the fold change in miRNA abundance between the reference group (0–7 days post-delivery, nonsmoking or nulliparous) and the exposed groups (7–14 days post-delivery, 14–30 days post-delivery, 30–80 days post-delivery, smoking, or multiparous) or per kg/m^2^ increase in BMI. All analyses were performed in the statistical program R (R Project for Statistical Computing, CRAN, The Comprehensive R Archive Network, Viena)^94^.

### miRNA pathway and ontology analysis

miRNAs from the top three highest expressing clusters underwent functional annotation with DIANA MirPATH version 3 online software (http://snf-515788.vm.okeanos.grnet.gr/)^95^. Two different mRNA target prediction algorithms (DIANA microT-CDS and TargetScan [using a high conservation score of 0.8]) were employed to identify putative mRNA targets of miRNAs. Experimentally validated targets were extracted from Tarbase v7.0, which is a database of over 500,000 experimentally validated mRNA-miRNA interactions. DIANA MirPath was then used to identify Kyoto Encyclopedia of Genes and Genomes (KEGG) pathways and gene ontology (GO) categories with significant enrichment (FDR ≤ 0.05). Hierarchical clustering was used to determine relationships between GO categories.

## Supplementary information

Supplementary Figures.

Supplementary Tables.

## Data Availability

The datasets generated during and/or analyzed during the current study are available on Gene Expression Omnibus (GEO) with Accession Number: GSE146880.
