# Supplementary material for: Human milk extracellular vesicle miRNA expression and associations with maternal characteristics in a population-based cohort from the Faroe Islands
Source: Sci Rep. 2021 Mar 12;11:5840. doi: 10.1038/s41598-021-84809-2 (PMC7970999; doi:10.1038/s41598-021-84809-2)
Supplement: Supplementary file 1 — Supplementary Figures. [file 41598_2021_84809_MOESM1_ESM.pdf]

Supplemental Figures

Human milk extracellular vesicle miRNA expression and associations with maternal characteristics in a population-based cohort from the Faroe Islands

Allison Kupsco[1]*, Diddier Prada[1,2], Damaskini Valvi[3], Lisa Hu[1], Pal Weihe[4,5], Brent Coull[7], Philippe Grandjean[6,7], Andrea A. Baccarelli[1]

[1]Department of Environmental Health Sciences, Columbia University Mailman School of Public Health, New York, NY 10023

[2]Unit for Biomedical Research in Cancer, Instituto Nacional de Cancerologia – Universidad Nacional Autonoma de Mexico, Mexico City, Mexico 14080

[3] Department of Environmental Medicine and Public Health, Icahn School of Medicine at Mount Sinai, New York, NY 10029, United States

[4]Department of Occupational Medicine and Public Health, The Faroese Hospital System, Tórshavn , Faroe Islands.

[5]Center of Health Science, University of The Faroe Islands, Tórshavn , Faroe Islands.

[6]Department of Environmental Medicine, University of Southern Denmark, Odense C , Denmark.

[7]Department of Environmental Health, Harvard T.H. Chan School of Public Health, Boston , MA, USA.

Corresponding Author:

Allison Kupsco
Email: ak4181@cumc.columbia.edu
P&S Building Room 16-416
630 W 168th St.
New York, NY 10032

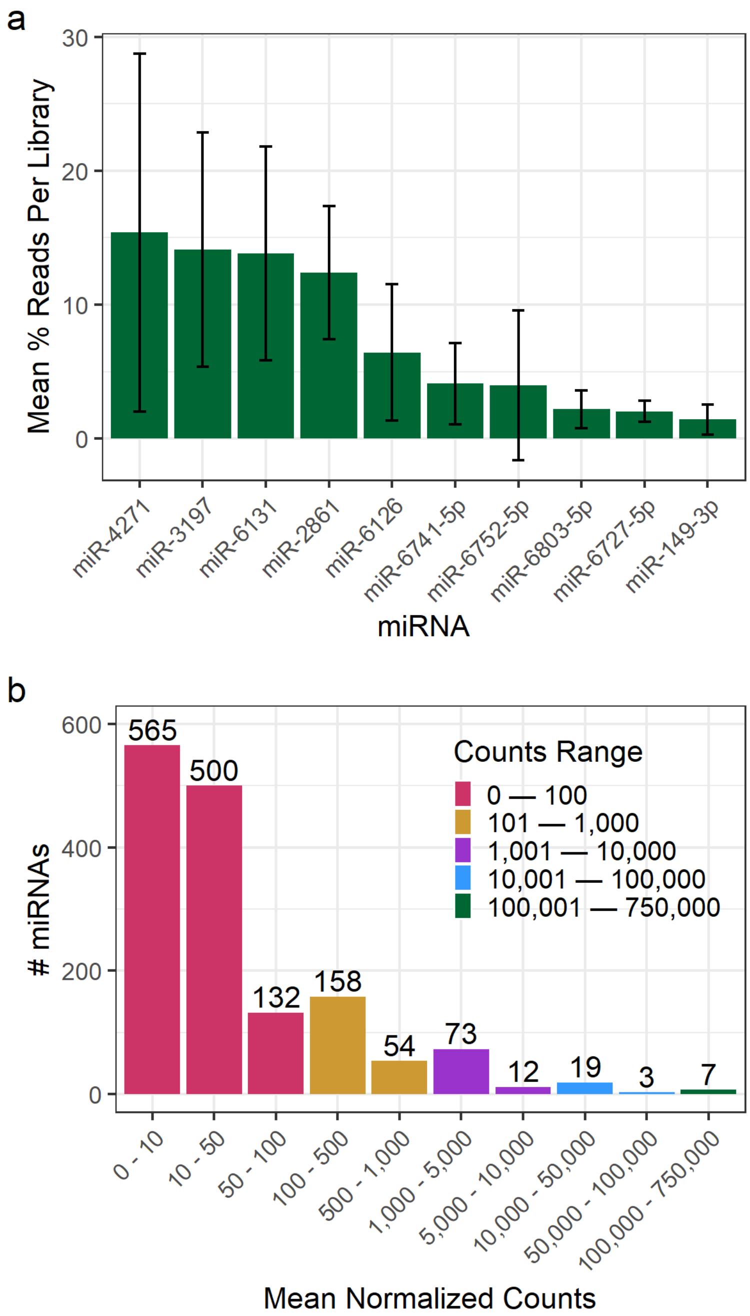

Supplemental Figure S1. Description of human milk extracellular miRNA (BMEV-miRNA) sequencing data (n=364). A) Average ± standard deviation of percentage reads per sequencing library for top 10 human milk EV-miRNAs. B) Histogram demonstrating the distribution of miRNAs with average expression (as normalized counts) ranging from zero to 750,000 counts per sample. Color indicates range bin: Pink: 0 – 100; Yellow: 101 – 1,000; Purple: 1,001 – 10,000; Blue: 10,001 – 100,000; Green 100,001 –750,000 average counts.

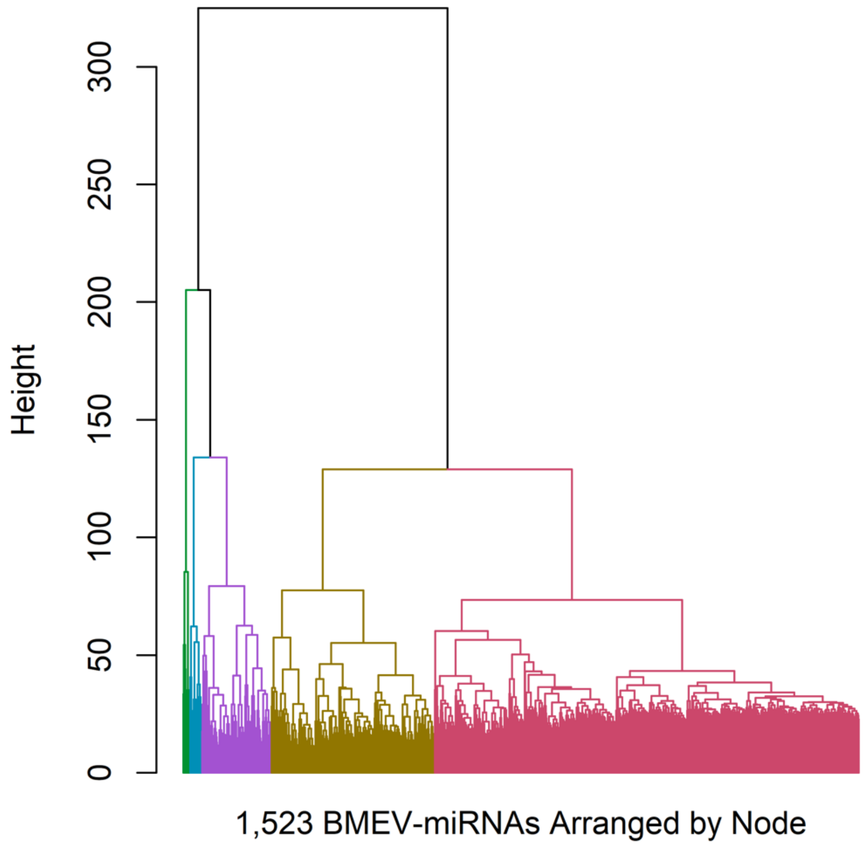

Supplemental Figure S2. Hierarchical clustering of human milk EV-miRNAs. Hierarchical clustering was performed with Euclidean distance and the complete linkage method to identified optimal miRNA clusters in 1,523 EV-miRNAs from breast milk. miRNAs are on the x-axis arranged by node and Euclidean distance is on the y-axis. Color of branches indicates cluster membership (Green: Cluster 1; Blue: Cluster 2; Purple: Cluster 3; Yellow: Cluster 4; Pink: Cluster 5).

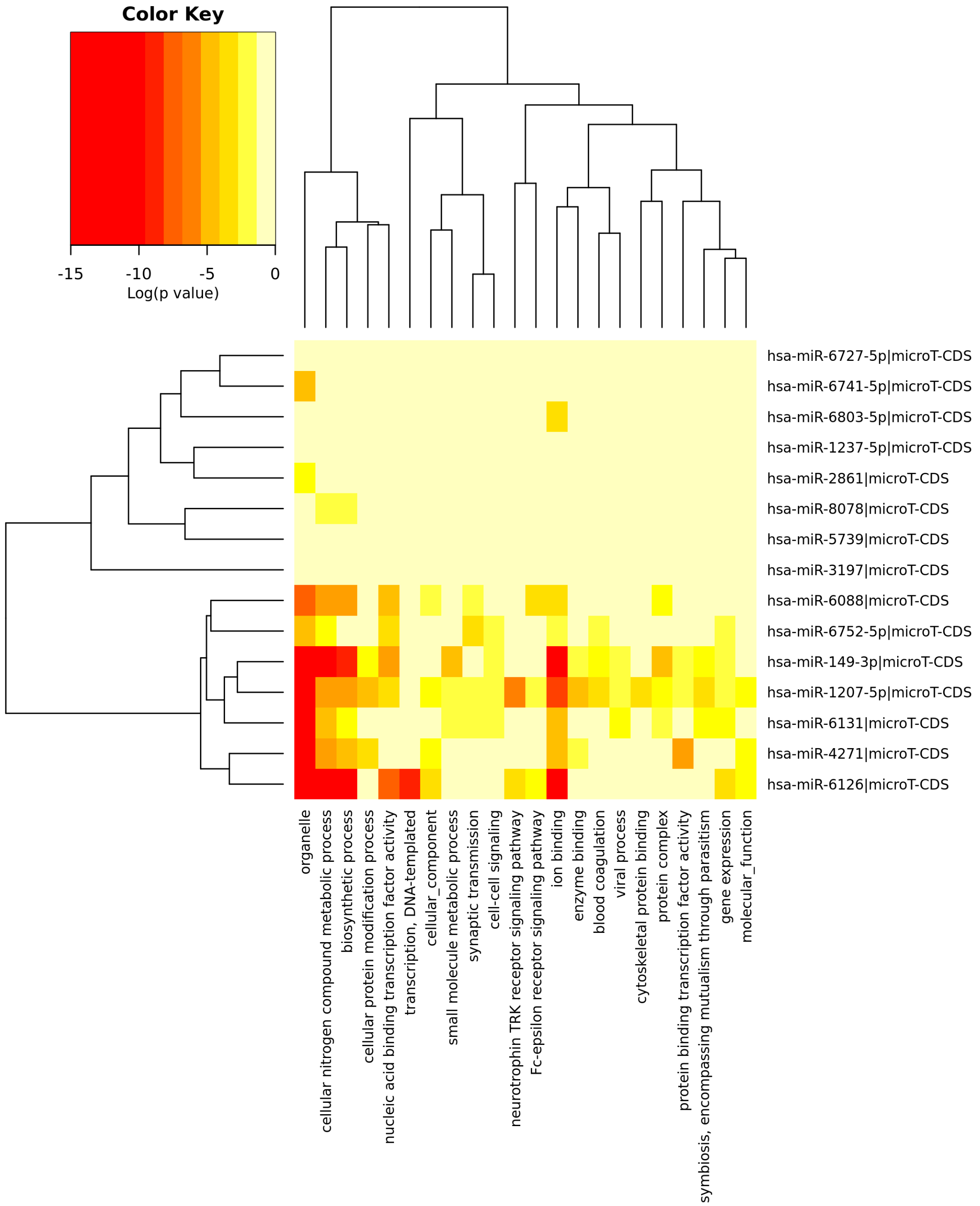

Supplemental Figure S3. Heatmap of False Discovery Rate (FDR) corrected log(p-values) from significant Gene Ontology (GO) categories from miRNAs from cluster 1. Gradient indicates strength of significance. DIANA microT-CDS version 3 (http://snf-515788.vm.okeanos.grnet.gr/) was used to predict miRNA mRNA targets and to generate the figure.

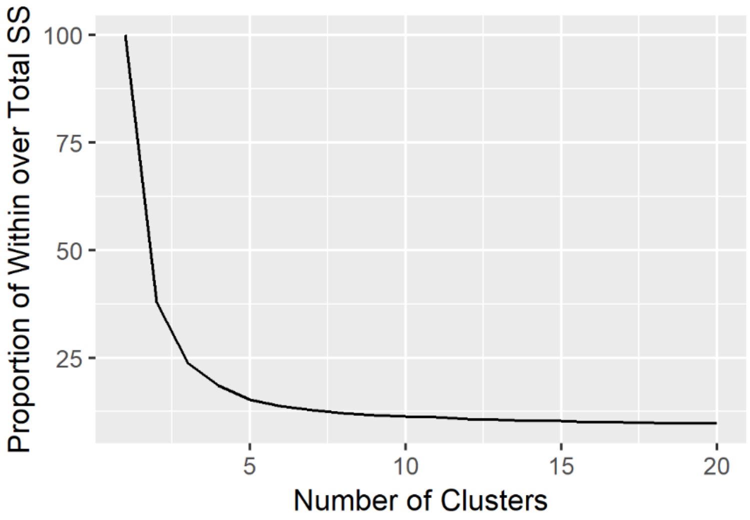

Supplemental Figure S4. K-means clustering to identify the optimal number of miRNA clusters in 1,523 BMEV-miRNAs. The proportion of within variance in comparison to the total sum of squares (SS) as the number of miRNA clusters increases.
